# Mechanistic insights into rumen function promotion through yeast culture (*Saccharomyces cerevisiae*) metabolites using *in vitro* and *in vivo* models

**DOI:** 10.3389/fmicb.2024.1407024

**Published:** 2024-07-16

**Authors:** Xue Chen, Jun Xiao, Wanzhu Zhao, Yanan Li, Wei Zhao, Weigang Zhang, Liang Xin, Zhiyi Han, Lanhui Wang, Natnael Demelash Aschalew, Xuefeng Zhang, Tao Wang, Guixin Qin, Zhe Sun, Yuguo Zhen

**Affiliations:** ^1^Key Laboratory of Animal Nutrition and Feed Science of Jilin Province, Key Laboratory of Animal Production Product Quality and Security Ministry of Education, JLAU-Borui Dairy Science and Technology R&D Center, College of Animal Science and Technology, Jilin Agricultural University, Changchun, China; ^2^Postdoctoral Scientific Research Workstation, Feed Engineering Technology Research Center of Jilin Province, Changchun Borui Science and Technology Co., Ltd., Changchun, China; ^3^College of Life Sciences, Engineering Research Center of Bioreactor and Pharmaceutical Development, Ministry of Education, Jilin Agricultural University, Changchun, China

**Keywords:** yeast culture metabolites, rumen, growth performance, slaughter performance, microorganism, lambs

## Abstract

**Introduction:**

Yeast culture (YC) enhances ruminant performance, but its functional mechanism remains unclear because of the complex composition of YC and the uncertain substances affecting rumen fermentation. The objective of this study was to determine the composition of effective metabolites in YC by exploring its effects on rumen fermentation *in vitro*, growth and slaughter performance, serum index, rumen fermentation parameters, rumen microorganisms, and metabolites in lambs.

**Methods:**

In Trial 1, various YCs were successfully produced, providing raw materials for identifying effective metabolites. The experiment was divided into 5 treatment groups with 5 replicates in each group: the control group (basal diet without additives) and YC groups were supplemented with 0.625‰ of four different yeast cultures, respectively (groups A, B, C, and D). Rumen fermentation parameters were determined at 3, 6, 12, and 24 h *in vitro.* A univariate regression model multiple factor associative effects index (MFAEI; y) was established to correlate the most influential factors on *in vitro* rumen fermentation with YC metabolites (x). This identified the metabolites promoting rumen fermentation and optimal YC substance levels. In Trial 2, metabolites in YC not positively correlated with MFAEI were excluded, and effective substances were combined with pure chemicals (M group). This experiment validated the effectiveness of YC metabolites in lamb production based on their impact on growth, slaughter performance, serum indices, rumen parameters, microorganisms, and metabolites. Thirty cross-generation rams (Small tail Han-yang ♀ × Australian white sheep ♂) with good body condition and similar body weight were divided into three treatment groups with 10 replicates in each group: control group, YC group, pure chemicals combination group (M group).

**Results:**

Growth performance and serum index were measured on days 30 and 60, and slaughter performance, rumen fermentation parameters, microorganisms, and metabolites were measured on day 60. The M group significantly increased the dressing percentage, and significantly decreased the GR values of lambs (*p*  < 0.05). The concentration of growth hormone (GH), Cortisol, insulin (INS), and rumen VFA in the M group significantly increased (*p* < 0.05).

**Discussion:**

These experiments confirmed that YC or its screened effective metabolites positively impact lamb slaughter performance, rumen fermentation, and microbial metabolism.

## 1 Introduction

The feed additives were used to manipulate the rumen microbial population when the ruminants under stressful conditions and rumen microflora gets disturbed (Vohra et al., 2016). To overcome these circumstances, *Saccharomyces cerevisiae* and its byproducts are commonly and widely used in animal production, especially yeast culture (YC) (Schlabitz et al., 2022). YC includes fermentation metabolites from aerobic and anaerobic processes in a specific medium. It comprises culture medium, cell metabolites, peptides, esters, sugars, organic acids, and growth-enhancing bioactive substances. Notably, YC may not contain viable yeast cells (Alzahal et al., 2014; Shurson, 2018). YC is rich in nutrients like amino acids, oligosaccharides, organic acids, and bioactive substances (β-glucan, mannan), which promotes animal growth (Gao et al., 2008; Peng et al., 2020). Liu et al. (2019a) reported YC supplementation in lambs, finding it improved growth and carcass traits. Lesmeister et al. (2004) also found that dairy calves fed starter with 2% YC showed increased dry matter intake, daily gain, and rumen development.

Yeast culture has a positive effect on the stabilization of ruminal pH and nutrient digestibility. This is mainly because YC can alter rumen fermentation by stimulating the growth and activity of cellulolytic bacteria (*Fibrobacter succinogenes* and *Ruminococcus albus*) and lactic acid-utilizing bacteria (*Selenomonas ruminantium*) (Callaway and Martin, 1997; Dias et al., 2018). Tun et al. (2020) showed dietary YC supplementation increased the abundance of *Ruminococcus flavefaciens*, *F. succinogenes*, *Prevotella brevis*, *R. flavefaciens*, ciliate protozoans, and *Bifidobacterium* spp. under both high (> 6.3) and low pH (5.8–6.0) conditions *in vitro*. Halfen et al. (2021) reported that dietary supplementation with YC promoted the abundance of fiber-digesting bacteria (*Eubacterium ruminantium* and *R. flavefaciens*) while decreasing amylolytic bacteria (*Ruminobacter amylophilus* and *Succinimonas amylolytica*) in the rumen, stabilizing rumen pH, and increasing volatile fatty acid (VFA) concentration. In addition, Longuski et al. (2009) found that YC supplementation had no significant effect on ruminal pH, VFA concentration, or the acetate: propionate ratio in the ruminal fluid when ruminal starch fermentability increased rapidly.

The composition of YC is extremely complex, and the functional substances in YC that affect rumen fermentation remain unclear, resulting in uncertainty surrounding their mechanisms of action. This has limited the scientific application of YC to some extent. Therefore, determining the effective metabolite groups and evaluating their effectiveness in YC are key problems that must be solved. Trail 1 aimed to produce different YCs that provide raw materials for identifying effective metabolites, and identify the optimal levels of the effective metabolites in YC. Trial 2 aimed to explore whether the effective substances combined with pure chemicals has a positive effect on lambs, so as to verify the effectiveness of the pure chemicals combination.

## 2 Materials and methods

### 2.1 Animal ethics

The animals were managed according to the guidelines for the care and use of experimental animals of Jilin Agricultural University Care and Use Committee and commercial dairy farms (JLAU-ACUC2021-013, Changchun, China).

### 2.2 Yeast culture preparation and metabolomic analysis

Yeast (*Saccharomyces cerevisiae*) was cultured in a 1 m^3^ fermenter for 10 h, moved to a 5 m^3^ fermenter for aerobic expansion culture, and finally transferred to a 20 m^3^ fermenter for anaerobic fermentation. The yeast grew on yeast peptone dextrose broth at an initial pH value of 6.0 and incubated at 30°C and 150 rpm/min for aerobic expansion culture, and incubated at 20°C and 50 rpm/min for anaerobic fermentation. After 6 h, 18 h, 36 h, and 48 h of anaerobic fermentation, the fermentation liquid (approximately 5 m^3^) was transferred to a homogenizer for wall-breaking treatment to produce different YCs. These were denoted as products A (YC 6 h), B (YC 18 h), C (YC 36 h), and D (YC 48 h). The main components of the YC were yeast extracellular metabolites, yeast cell contents, broken yeast cell walls, inactivated yeast cells, and mutated culture medium. Yeast culture products used in this study were provided by Changchun Borui Technology Co., Ltd. (Changchun, China).

Different YC samples (15 mL) with five replicates were dried using a freeze drier (Alpha1-2LDplus, Martin Christ Gefriertrocknungsanlagen GmbH, Osterode am Harz, Germany) at −45°C for 36 h for metabolites analysis. The dried samples were accurately weighed (0.05 g) into 1.5 mL EP tubes for derivatization with bis (trimethylsilyl)-trifluoroacetamide (BSTFA) following a previous study (Chen et al., 2019). The samples were then subjected to detection via gas chromatography–mass spectrometry (GC/MS; Trace1300-ISQ7000, Thermo Fisher Scientific, Waltham, MA, United States) with an HP-5MS capillary column (30 m × 0.25 mm i.d. × 0.25 μm). The initial temperature of the column was kept at 80°C for 3 min, increased to 150°C at a rate of 5°C/min for 10 min, and further increased to 280°C at a rate of 10°C/min. The column was then maintained at this temperature for 10 min. The solvent delay time was 3 min, and the shunt ratio was 10:1. Helium was used as a carrier gas at a flow rate of 1 mL/min. The energy was 70 eV in the electron impact mode. The temperatures of the ion source and transfer line were 230°C and 280°C, respectively. The MS spectra were acquired from a mass-to-charge ratio (m/z) range of 20–800 in full-scan mode. Metabolites were identified via probability-based matching of their mass spectra with National Institute of Standards and Technology, and the minimum matching similarity was set to 800. BSTFA was then removed to obtain the final metabolites.

### 2.3 Trial 1: screening for effective metabolites in yeast culture

#### 2.3.1 Collection of ruminal fluid

Three male small-tail Han-yang lambs (body weight: 50 ± 2 kg; age: 12 ± 1 months) equipped with permanent rumen cannulas were selected. Animals were kept in individual rearing cages and were fed mixed pellet diet (Table 1) twice at 8:00 a.m. and 5:00 p.m. The lambs were given free access to clean water. Mixed rumen fluid was collected from the bottom, middle, and back areas of the rumen using a rigid polyvinyl chloride tube 2 h before morning feeding. About 500 mL of rumen fluid was collected from each lamb. The mined rumen fluid was mixed and put into a thermos preheated to 39°C filled with CO_2_ and returned to the laboratory quickly.

**Table 1:** Ingredients and nutritional composition of the experimental diet.

Ingredients (%)		Nutritional components (% dry matter)	
Corn	35.00	Crude protein	14.27
Soybean meal	13.00	Neutral detergent fiber	28.17
Corn germ meal	6.00	Acid detergent fiber	18.35
Corn bran	10.00	Starch	25.87
Peanut hull	23.00	Ether extract	3.94
Bentonite	4.00		
Sucrose	4.00		
^a^Premix	5.00		
Total	100.00		

a Premix composition (per kilogram): Ca^2+^110 g, Cu^2+^ 140 mg, Zn^2+^ 930 mg, Mn^2+^ 600 mg, Co^2+^ 13 mg, I^−^ 20 mg, Se^4+^ 13 mg, vitamin A 340 KIU, vitamin D_3_ 120 KIU, vitamin E 1700 IU.

#### 2.3.2 Experimental design

A single-factor design was used, and the experiment was divided into five treatment groups with 5 replicates in each group. The control group (basal diet without additives, Table 1) and four YC groups were supplemented with 0.625‰ (the optimal dosage determined by the previous test) of four different YCs (YC-A, group A; YC-B, group B; YC-C, group C; YC-D, group D). An ANKOM radio frequency gas production system was used in this experiment. Samples (2 g) were weighed in a nylon bag (400 mesh, 6 cm × 7 cm). The bags were sealed using a heat sealer (model #AIE-200, American International Electric, City of Industry, CA, United States) and placed inside 250 mL sample bottles. Each bottle was filled with 40 mL rumen fluid and 80 mL buffer, which was prepared anaerobically as described by Menke and Steingass (1988). Subsequently, the bottles were filled with CO_2_ gas for 30 s, ensuring the lid was securely closed before culturing in a gas bath incubator at 39°C with a rotation speed of 80 rpm.

#### 2.3.3 Determination of fermentation parameters

After culturing for 3, 6, 12, and 24 h, five sample bottles of each group were removed and cooled by placing into ice water (4°C) to terminate the fermentation. The nylon bags were then removed, rinsed in a washing machine for 10 min at medium speed, and dried in an oven at 65°C until a constant weight was achieved. Finally, dry matter (DM) was determined, and DM degradability (DMD) was calculated as follows (Chen and Yu, 2020) (Eq. 1):


(1) $$DMD\;\left(\%\right)=\frac{A-\left(B-W\times K\right)}{A}\times 100$$

where A denotes the sample weight before *in vitro* fermentation (DM basis), B denotes the sample weight after *in vitro* fermentation plus the nylon bag weight (DM basis), W denotes the nylon bag weight after *in vitro* fermentation (weight after drying at 105°C), and K denotes the nylon bag correction (weight after drying at 105°C after *in vitro* fermentation/weight before *in vitro* fermentation).

The pH of the fermentation liquid was immediately measured using a pH meter (Sanxin MP523-04; Shanghai Sanxin Instrumentation, Inc., Shanghai, China). Subsequently, we stored 5 mL and 8 mL samples at −40°C for the subsequent determination of ammonia nitrogen (NH_3_-N) and microprotein (MCP), respectively. Analyses of the NH_3_-N was performed according to the method described by Chaney and Marbach (1962), and MCP was measured using the method by Zinn and Owens (1986) with an ultraviolet spectrophotometer (SHIMADZU UVmini-1280, Shimadzu, Kyoto, Japan).

A total of 1 mL of the sample after centrifugation (10,000 r/min, 10 min) was mixed with 200 μL metaphosphoric acid (concentration: 25%) and stored at −40°C for the determination of VFA (Liu et al., 2019a). VFAs were analyzed using an Agilent 7890B Gas Chromatograph (Agilent Technologies, Santa Clara, CA, United States) with a flame ionization detector and a capillary column (30 m × 0.32 mm i.d. × 0.50 μm). Helium was used as a carrier at a constant flow rate. The initial temperature of the oven was 65°C, which was steadily increased to 190°C at a rate of 20°C/min. The temperature of the detector and injection was 250°C, and the injection volume of the sample was 1 μL.

#### 2.3.4 Calculation of associative effects

Single-factor associative effects index (SFAEI) and MFAEI were determined according to a previous study (Yuan and Xinjie, 2019) (Eq. 2):


(2) $$\mathrm{SFAEI}=\frac{\overset{n}{\underset{n=1}{\sum }}A2-A1}{n\times A3}$$

where A1 corresponds to DMD (%), the concentration of NH_3_-N, total VFA (acetic acid, propionic acid, isobutyric acid, butyric acid, isovaleric acid, and valeric acid), and MCP at different *in vitro* fermentation times in the control group. A2 represents the DM degradation rate, concentration of NH_3_-N, total VFA, and MCP at different *in vitro* fermentation times in different YC groups. A3 denotes the average summation of A2 at different *in vitro* fermentation times, and *n* denotes the number of *in vitro* fermentations.


$$\begin{array}{c} \mathrm{SFAEI}=\Sigma {\mathrm{SFAEI}}_{\left(DMD\%\right)}+\Sigma {\mathrm{SFAEI}}_{\left(NH3-N\right)}+\Sigma {\mathrm{SFAEI}}_{\left(\mathrm{TVFA}\right)}\\ +\;\Sigma {\mathrm{SFAEI}}_{\left(MCP\right)}\text{,} \end{array}$$

MFAEI=SFAEI

### 2.4 Trial 2: validation of the effective metabolites in different yeast cultures for lamb production tests

#### 2.4.1 Experimental design and animal feed management

Thirty cross-generation rams (small-tail Han-yang ♀ × Australian white sheep ♂) with good body condition and similar body weight (33.51 ± 2.36 kg) and age (3 months old) were selected. After 14 d of adaptation, the lambs were weighed on the first day of the formal trial period. To maintain similar body weights (34.69 ± 1.74 kg, *p* > 0.05) among the 30 lambs, a single-factor experimental design was employed, randomly dividing them into three treatment groups: the control group (basal diet with no additives), YC group (basal diet supplemented with 0.625‰ product B), and pure chemicals combination group (M group, basal diet supplemented with 0.037‰ pure product). The lambs were fed in individual cages with fecal leakage plates and were dewormed and vaccinated during the transition period. The test animals were fed twice per day, at 8:00 a.m. and 5:00 p.m., and the remaining feed was weighed before the morning feeding session. Daily feeding amounts were adjusted based on the lambs’ actual feed intake to ensure *ad libitum* feeding and access to clean drinking water. This feeding regimen was continued for 60 d.

Animals were fed a mixed diet (pellet diet) and the diet formula was the same as in trail 1 (Table 1). All animals were fed a basal diet during the transitional period, and the animals were fed an experimental diet during the formal trial period according to their respective treatments. Following Trial 1, it was provisionally considered that metabolites with no significant positive correlation with MFAEI in YC amounted to 0%, and the identified effective metabolites from pure chemicals were combined. The supplementation of YC during rumen fermentation *in vitro* was 0.625‰ in Trial 1, and the added amount of the effective metabolites in YC was equivalent to 0.037‰. To ensure the same content of effective metabolites in YC and pure chemical combination, the supplementation of the pure chemical combination was 0.037‰.

#### 2.4.2 Sampling and analysis

The live weight of each lamb was recorded before the morning feeding session on days 1 and 60 of the formal trial period. Dry matter intake (DMI) was recorded daily, and the average daily gain (ADG) was calculated.

Fasting blood samples from each lamb in each treatment group were collected through the jugular vein using 10 mL vacuum blood collection tubes on the morning of days 1, 30, and 60. The blood samples were left undisturbed for 4–5 h to separate the serum, and then centrifuged at 3,000 rpm for 10 min at 4°C. Serum was subsequently collected and stored at −40°C for future analysis. Serum levels of growth hormone (GH), cortisol, insulin (INS), and leptin (LEP) were analyzed using a commercial sheep enzyme-linked immunosorbent assay kit (Shanghai Enzyme-Linked Biotechnology Co. Ltd., Shanghai, China; Dong et al., 2022; Zhao et al., 2024). Quantitative colorimetric kits (Nanjing Jiancheng Bioengineering Institute, Nanjing, China) were used to determine the concentration of glucose (GLU, Vieira et al., 2010). TP was measured using the colorimetric and biuret technique, and ALB level was measured using the colorimetric and bromocresol green method (Ramella et al., 2020).

All animals were slaughtered on day 61 or 62. Lambs were slaughtered every 30 min and the water and feed troughs were emptied 6 h before one lamb slaughter. The lamb was packed into a single cage with wheels for weighing, and then transported to a slaughter room 50 meters. The slaughtering process was carried out by a professional to reduce experimental error. The carcass, fur, head, hooves, testes, and abdominal fat were weighed after slaughter, and the percentages of slaughter and abdominal fat were calculated. The tissue thickness (GR, mm) between the twelfth and thirteenth ribs was accurately measured using a Vernier caliper 11 cm from the midline of the ridge. Approximately 100 g of chyme was collected from the upper, middle, and lower parts of the rumen and mixed in two sterile test tubes for the analysis of VFA (Liu et al., 2019a), rumen microorganisms, and metabolites.

Dressing percentage was calculated as follows: carcass weight divided by live weight before slaughter, multiplied by 100%. Abdominal fat percentage was determined by dividing the total fat weight of the abdominal mesentery and greater omentum in the carcass by the carcass weight.

### 2.5 Rumen microorganisms

Total DNA was extracted using a Fast DNA SPIN extraction kit (MP Biotechnology, CA, United States), and DNA concentration was measured using a NanoDrop (ND-1000, Thermo Fisher Technology, MA, United States). The V3–V4 regions of the 16S rRNA genes were amplified using the universal primers 338F (5′-ACTCCTACGGGAGGCAGCA-3′) and 806R (5′-GGACTACHVGGGTWTCTAAT-3′). The amplification system and conditions were as previously described by Dill-McFarland et al. (2017). VAHTSTM DNA Clean Beads (Vazyme, Nanjing, China) and a PicoGreen dsDNA Assay Kit (Invitrogen, Carlsbad, CA, United States) were used to purify and quantify the PCR products. The purified PCR products were pooled and sequenced using the Illumina MiSeq platform. Sequencing data were analyzed using Quantitative Insights into Microbial Ecology (QIIME) 2 software^1^ (Bolyen et al., 2019). Sequences were then quality filtered, denoised, and merged, and chimeras were removed using the DADA2 plugin (Callahan et al., 2016). The sample sequence was normalized based on the ASV, and we selected the average maximum flattening depth score to compute alpha diversity indices, including Chao1, Faith_pd, and Shannon. A sparse curve was generated to visualize the data. Unweighted UniFrac distance, non-metric multidimensional scaling analysis (NMDS), and analysis of similarity (ANOSIM) (Lozupone et al., 2005) were applied using QIIME 2 (“vegan” package in R) to calculate the distance matrix of each sample, analyze the β-diversity of different samples, and analyze the differences between the groups.

### 2.6 Rumen metabolites

The samples were centrifuged at 4,000 rpm at 4°C for 10 min, and the supernatant was collected and freeze-dried at −45°C for 48 h. For the drying process, the samples were meticulously weighed (0.01 g) into 1.5 mL EP tubes for silylation and then underwent silanization and derivatization. The methods of derivatization and determination through GC/MS have been described in section 2.2.

### 2.7 Data analysis

Data were statistically analyzed via one-way analysis of variance using SPSS software. We conducted Pearson correlation analysis to examine the relationship between the relative concentrations of metabolites in YCs and MFAEI, considering *p* < 0.05 as indicative of a significant difference. Metabolites with significant positive correlations (R > 0 and *p* < 0.05) (x_1_, x_2_, x_3_……x_i_) and MFAEI (y) were selected to establish a univariate regression equation (*y = f (xi)*). The models in Table 2 were applied.

**Table 2:** Univariate regression models.

Model	Equation
Linear equation	y = b_0_ + b_1_x
Quadratic equation	y = b_0_ + b_1_x + b_2_x^2
Compound curve equation	y = b_0_ b_1_ × ^2
Growth curve equation	y = e^(b_0_ + b_1_ x)
Exponential curve equation	y = a^x^
Logistic curve equation	y(t) = 1/(1 + x^−t^)

The screened rumen metabolite data were analyzed using SIMCA 14.0 software (Umetrics AB, Umeå, Sweden). Principal component analysis (PCA) was performed to visualize the distribution and separation trends of rumen metabolites among different treatments. Orthogonal partial least squares-discriminate analysis (OPLS-DA) models were used to validate the model against overfitting. Both the variable importance in the projection (VIP > 1) values and Student’s *t*-test (*p* < 0.05) were used to determine the significant metabolites between the control and YC groups, and between the control and M groups. Candidate metabolites were analyzed using Metaboanalyst 4.0^2^ for path enrichment and topology analysis. Kyoto Encyclopedia of Genes and Genomes (KEGG; http://www.Genome.jp/kegg/) was used to assess the significant metabolic pathways.

## 3 Results

### 3.1 Trial 1

#### 3.1.1 Metabolites of different yeast cultures

**Figure 1:**
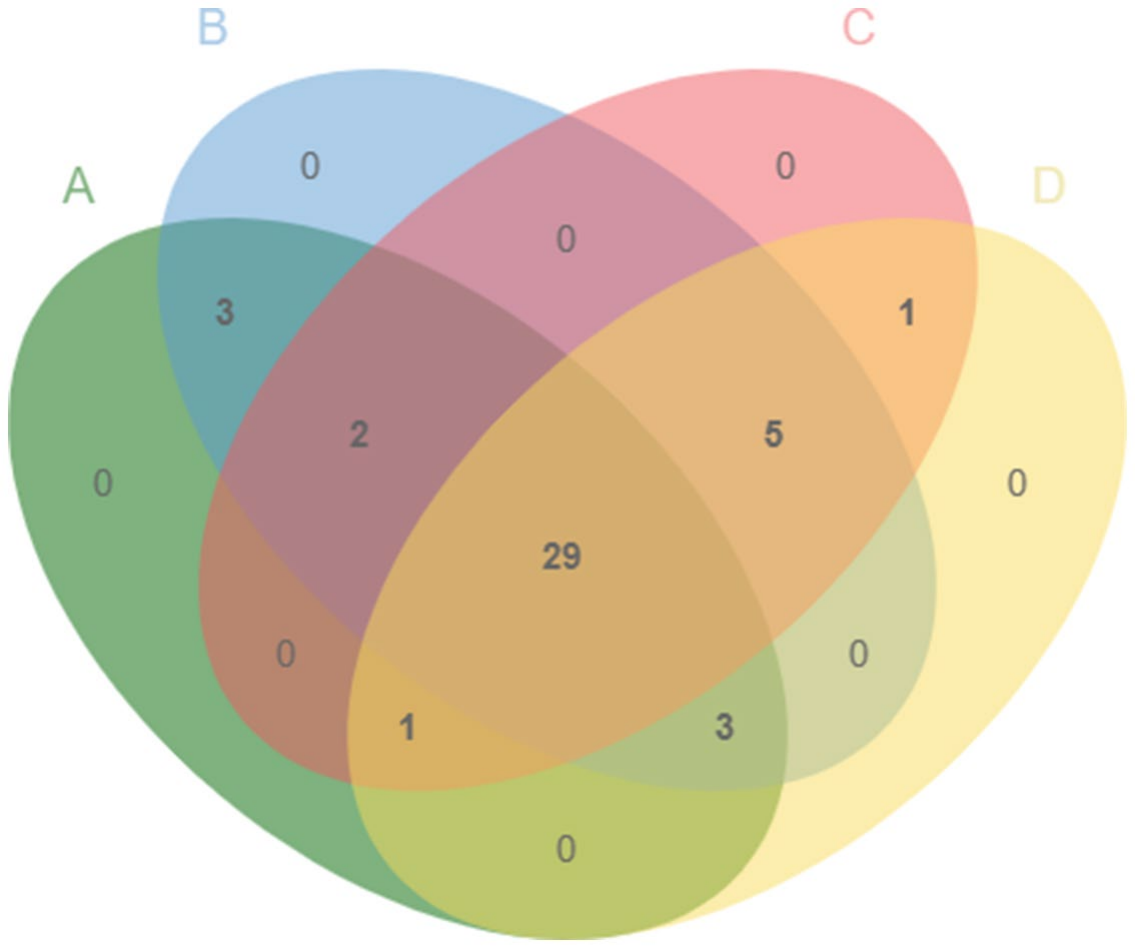
Venn diagram of the metabolites in products A, B, C, and D.

A total of 40 metabolites in 4 different YC products were identified by GC/MS after removing the components of the culture medium and derivatives (Figure 1). Metabolites of YC mainly included carbohydrates, organic acids, fatty acids, alcohols, and amino acids. There were 29 common metabolites among the 4 different YC products. 3-A-mannobiose, gentiobiose, and maltose were the common metabolites of products A and B; propanoic acid and 5-hydroxytryptophan (5-HTP) were the common metabolites of products A, B and C; and ethanamine, ethane, isocyanato, L-valine, pentanamide, and L-isoleucine were the common metabolites of products B, C, and D.

#### 3.1.2 Rumen fermentation parameters *in vitro*

**Figure 2:**
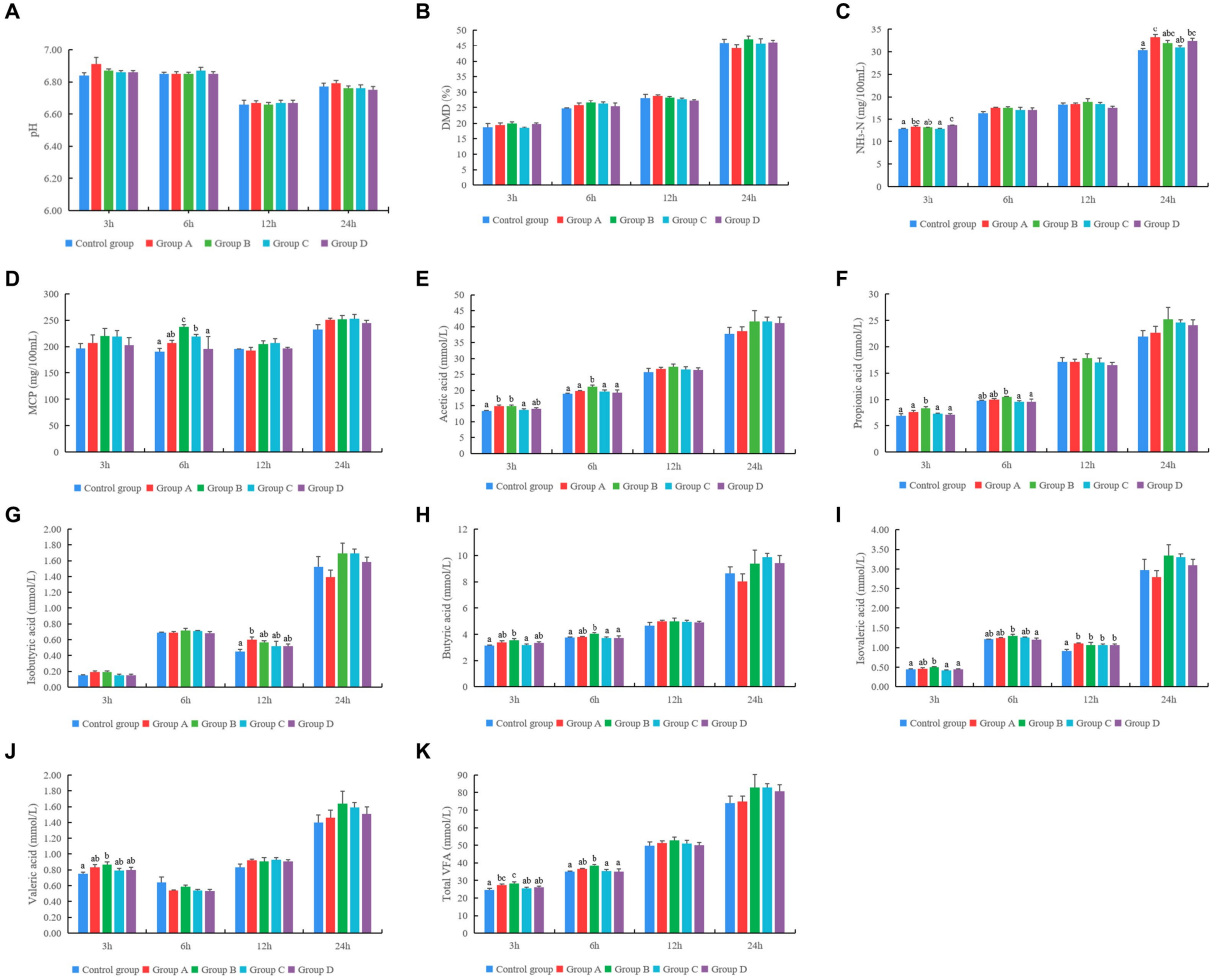

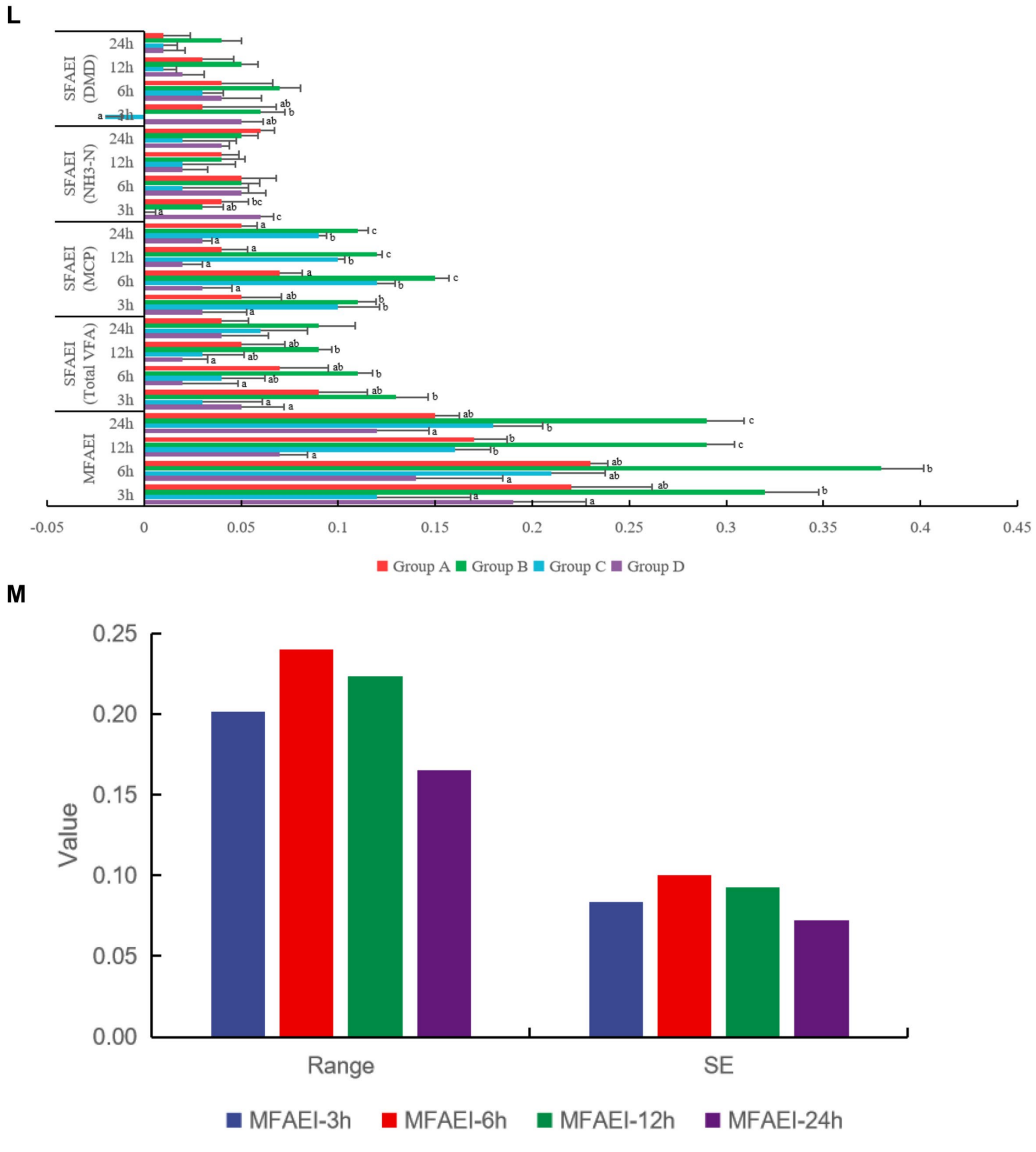
Effect of different yeast culture (YC) on **(A–K)** rumen fermentation parameters, **(L)** single-factor associative effects index (SFAEI) and multiple factor associative effects index (MFAEI), and **(M)** range mean MAX_(MFAEI)_-MIN_(MFAEI)_ and SE means standard error at different rumen fermentation *in vitro*. Different letters indicate significant differences (*p* < 0.05); this is valid for all subsequent figures in this article.

Figures 2A–D shows that different YC products had no significant effect on pH and dry matter degradation rate at different times of rumen fermentation *in vitro* (*p* > 0.05). The concentration of NH_3_-N in groups A and D was significantly higher than that of the control group at 3 and 24 h of rumen fermentation *in vitro*. The concentration of MCP in groups B and C was significantly higher than that in the control group at 6 h of rumen fermentation *in vitro* (*p* < 0.05) (Figures 2E–K).

The concentration of acetic acid in groups A and B was significantly higher than that in the control group at 3 h. Furthermore, *in vitro* acetic acid concentration at 6 h showed the highest value in group B (*p* < 0.05). The concentration of propionic acid in group B was significantly higher than that in other groups at 3 h and significantly higher than that in groups C and D at 6 h of fermentation times (*p* < 0.05). The concentration of isobutyric acid in group A was significantly higher than that in the control group at 12 h *in vitro* (*p* < 0.05). The butyrate concentration in group B was significantly higher than that in the control group at 3 and 6 h (*p* < 0.05). The concentration of isovaleric acid in group B was significantly higher when compared with that of the control group at 3 h. Further, the concentration of isovaleric acid in groups supplemented with YC was significantly higher than that of the control group at 12 h of rumen fermentation *in vitro* (*p* < 0.05). The concentration of valerate in group B was significantly higher than that in the control group at 3 h of rumen fermentation *in vitro*. The total VFA concentration in groups A and B was significantly higher than that of the control group on 3 h (*p* < 0.05). The total VFA concentration in group B was significantly higher when compared with that of the control group at 6 h of rumen fermentation *in vitro* (*p* < 0.05) (Figures 2E–K).

According to the results of the MFAEI, product B showed the best effect on rumen fermentation at different fermentation times *in vitro* (Figure 2L). At 3, 6, 12, and 24 h, the variation degree of MFAEI among the YC groups was different, and the variation degree at 6 h was greater than that at 3, 12, and 24 h of rumen fermentation *in vitro* according to the range and standard error of MFAEI among YC groups (Figure 2M). At 6 h, the range and standard error of MFAEI among YC groups were 0.32 and 0.13, respectively. Therefore, the different YC showed the greatest effect at 6 h of rumen fermentation *in vitro*, so the effect of different YC was evaluated at 6 h of rumen fermentation *in vitro*.

#### 3.1.3 Correlation analysis of different YC products and MFAEI

**Figure 3:**
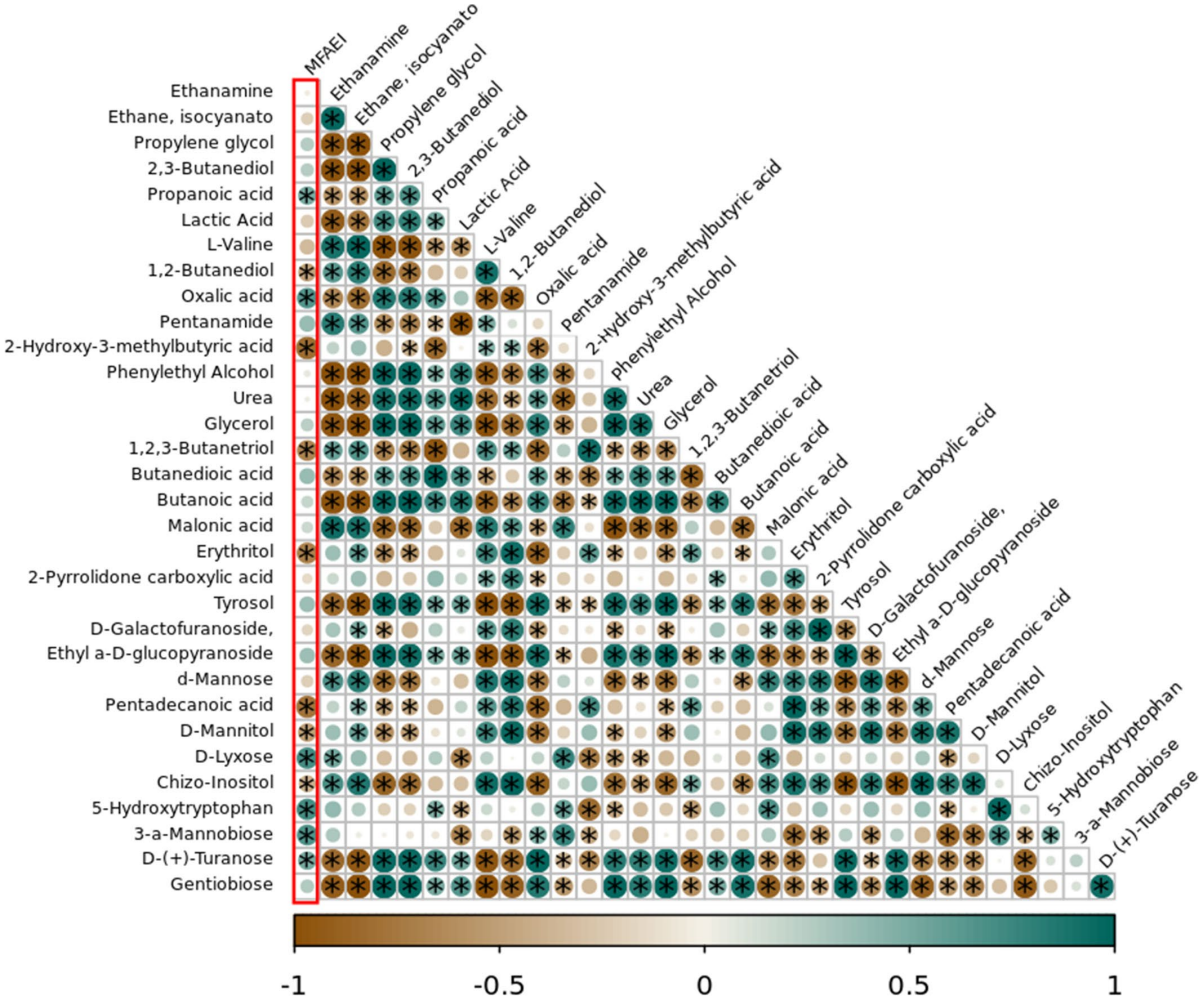
Correlation analysis between different yeast cultures and multiple factor associative effects index (MFAEI). The yeast metabolites marked with * in green circles of the first column were significantly positively correlated with MFAEI.

The Pearson correlation results between the metabolites in different YC products and MFAEI at 6 h of rumen fermentation *in vitro* showed that the relative concentrations of propanoic acid, oxalic acid, D-lyxose, 5-HTP, 3-a-mannobiose, and D-(+)-turanose metabolites were significantly positively correlated with MFAEI (*p* < 0.05) (Figure 3). Conversely, 1, 2-butanediol, 2-hydroxy-3-methylbutyric acid, 1,2, 3-butanetriol in YC products, erythritol, pentadecanoic acid, D-mannitol, and chizo-inositol were negatively correlated with MFAEI (*p* < 0.05). Therefore, the effective substances in YC that promote rumen fermentation were preliminarily determined to be the aforementioned six metabolites with significant positive correlation with MFAEI.

#### 3.1.4 Model establishment

The MFAEI of different YCs after 6 h of *in vitro* rumen fermentation was designated as “y”, corresponding to the six effective substances (propanoic acid, oxalic acid, D-lyxose, 5-hydroxytryptophan, 3-a-mannobiose, and D-(+)-turanose) in different YCs (A, B, C, and D), and their relative concentrations as x1, x2, x3, x4, x5, and x6, respectively. A univariate regression model, *y = f (xi)*, was established between MFAEI and each effective substance. The models applied are as followed.

Among the univariate regression models correlating MFAEI with YC metabolites, the quadratic equation models showed the correlation coefficients closest to 1 and the smallest *p*-values. The equations are shown in Table 3.

**Table 3:** Equations between multiple factor associative effects index and each effective substance.

Metabolite	Equation	Maximum value
propanoic acid, x_1_	y = −0.252x_1_^2^ + 0.386x_1_ + 0.047	x_1_ = 0.77
oxalic acid, x_2_	y = −0.529x_2_^2^ + 0.694x_2_−0.051	x_2_ = 0.66
D-lyxose, x_3_	y = −0.324x_3_^2^ + 0.53x_3_−0.03	x_3_ = 0.82
5-hydroxytryptophan, x_4_	y = −0.846x_4_^2^ + 0.669x_4_ + 0.05	x_4_ = 0.40
3-a-mannobiose, x_5_	y = −0.416x_5_^2^ + 0.525x_5_ + 0.048	x_5_ = 0.63
D-(+)-turanose, x_6_	y = −0.025x_6_^2^ + 0.168x_6_−0.098	x_6_ = 3.36

Using the equations in Table 3, the optimal content levels of the six effective substances in YC were determined as follows: x_1_ = 0.77%, x_2_ = 0.66%, x_3_ = 0.82%, x_4_ = 0.40%, x_5_ = 0.63%, and x_6_ = 3.36% when maximizing the Y value, i.e., when MFAEI is at its peak. After disregarding metabolites in YC that exhibited a negative or non-significant positive correlation with MFAEI, and combining the six effective substances with pure chemicals, the quantitative ratio among these six active substances was found to be propanoic acid: oxalic acid: d-lyxose: 5-hydroxytryptophan: 3-a-mannobiose: D-(+) -turanose = 11.56:9.90:12.34:5.97:9.52:50.71. In product B, the contents of these six effective metabolites were 0.60, 0.66, 0.66, 0.55, 0.88, and 2.60%, respectively.

### 3.2 Trial 2

#### 3.2.1 Growth and slaughter performance of lambs

Table 4 shows the growth performance of lambs in different treatments. Compared with the control group, there were no significant differences in DMI, ADG, and the ratio of feed to gain in the YC and M groups (*p* > 0.05). Dietary supplementation with YC or pure chemicals had a positive effect on the growth performance of lambs, and the ADG in the YC and M groups was increased by 8.15% compared with the control group. The dressing percentage of the M group was significantly higher than those of the control and YC groups (*p* < 0.05). The GR value of the M group was significantly lower than that of the control group (*p* < 0.05). There were no significant differences in abdominal fat percentage, fur + testis, head, and hooves weight among the treatments (*p* > 0.05).

**Table 4:** Effects of yeast culture (YC) and pure chemicals combination (M) on growth and slaughter performance of lambs.

Item	Control group	YC group	M group	SE	*p*-value
DMI (kg/d)	1.71	1.85	1.88	0.04	0.10
ADG (kg/d)	0.27	0.29	0.29	<0.01	0.29
Ratio of feed to gain, F/G	6.44	6.374	6.47	0.12	0.77
Dressing percentage (%)	44.47^a^	45.11^a^	48.23^b^	0.51	<0.05
GR value (mm)	10.69^b^	9.90^ab^	9.33^a^	0.29	<0.05
Abdominal fat percentage (%)	4.55	4.65	3.88	0.24	0.24
Fur + Testicles (kg)	8.95	8.85	8.15	0.19	0.10
Hoof (kg)	1.48	1.48	1.42	0.02	0.28
Head (kg)	3.30	3.40	3.00	0.11	0.15

#### 3.2.2 Serum index of lambs

**Figure 4:**
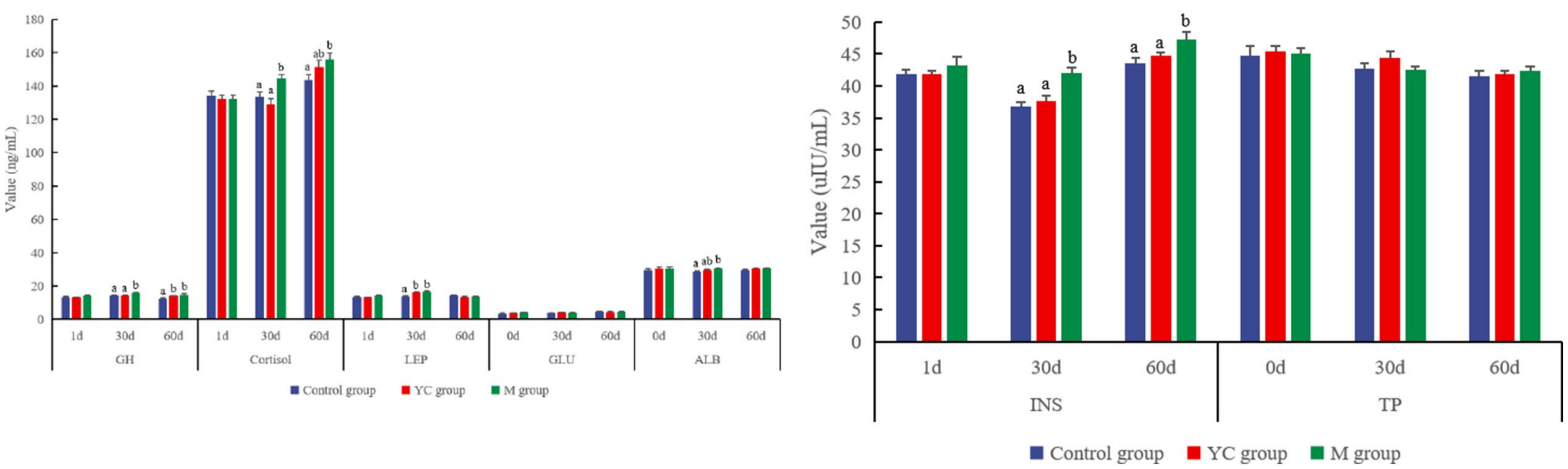
Effects of combined yeast culture (YC) and pure chemicals (M) on the serum index of lambs.

There was no significant difference in the serum hormone levels among the treatments on day 1 (*p* > 0.05, Figure 4). The level of GH in serum in the M group was significantly higher than that in the control and YC groups on day 30, and the GH in the YC and M groups was significantly increased by 11.10 and 17.01%, respectively, when compared with the control group on day 60 (*p* < 0.05). The concentration of cortisol in serum in the M group was significantly higher than that of the control and YC groups on day 30 (*p* < 0.05) and that of the control group on day 60 (*p* < 0.05). Compared with the control group, the concentration of INS in the YC group showed an increasing trend, while the INS level in the M group was significantly higher than that of other groups on days 30 and 60 (*p* < 0.05). The concentration of LEP in the YC and M groups was significantly higher than that in the control group on day 30 (*p* > 0.05). YC and the pure chemicals combination had no significant effects on the blood GLU and TP of sheep (*p* > 0.05). The concentration of ALB in the M group was significantly increased compared to that in the control group on day 30 (*p* < 0.05).

#### 3.2.3 Rumen fermentation of lambs

**Figure 5:**
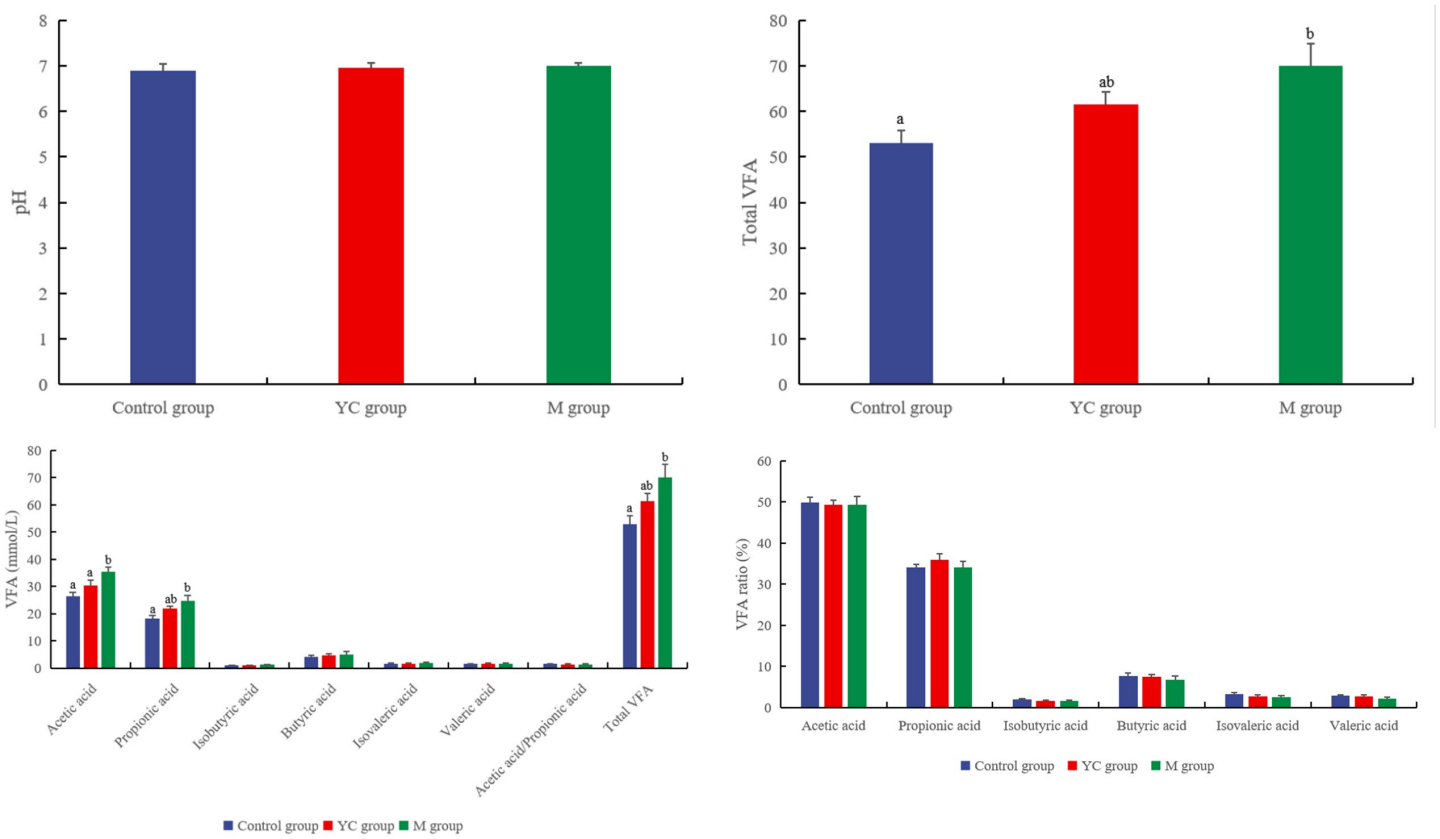
Effects of combined yeast culture (YC) and pure chemicals (M) on rumen fermentation of lambs.

There was no significant difference in rumen pH values among all groups at 6 h after feeding (*p* > 0.05, Figure 5). The concentration of acetic acid in the M group was significantly higher than that in the control and YC groups (*p* < 0.05). The concentrations of propionic acid and total VFA in the M group were significantly higher than those in the control group (*p* < 0.05). The diet combining YC and pure chemicals had no significant effects on the proportions of acetic, propionic, isobutyric, butyric, isovaleric, and valeric acids (*p* > 0.05).

#### 3.2.4 Rumen microorganisms

**Figure 6:**
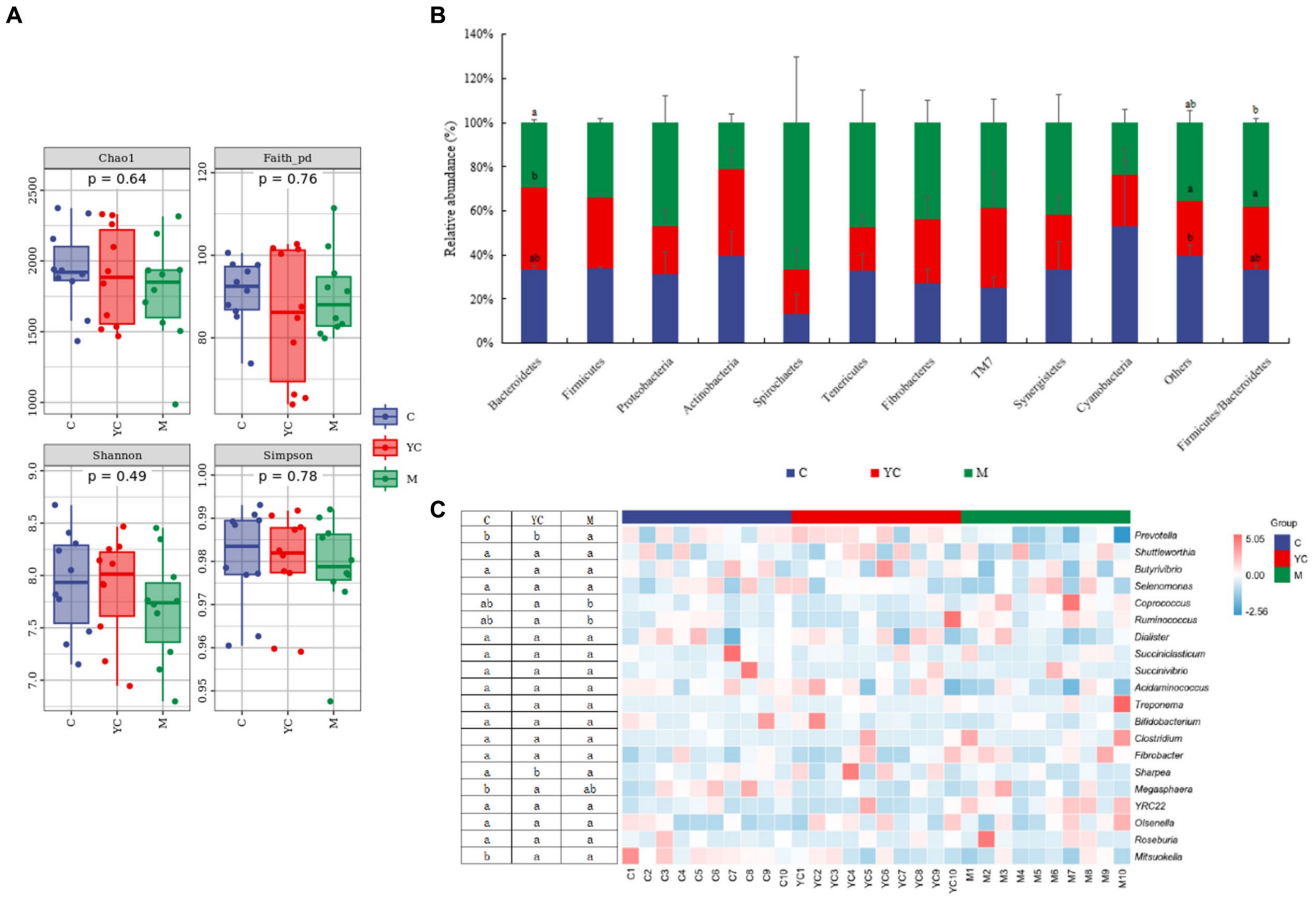

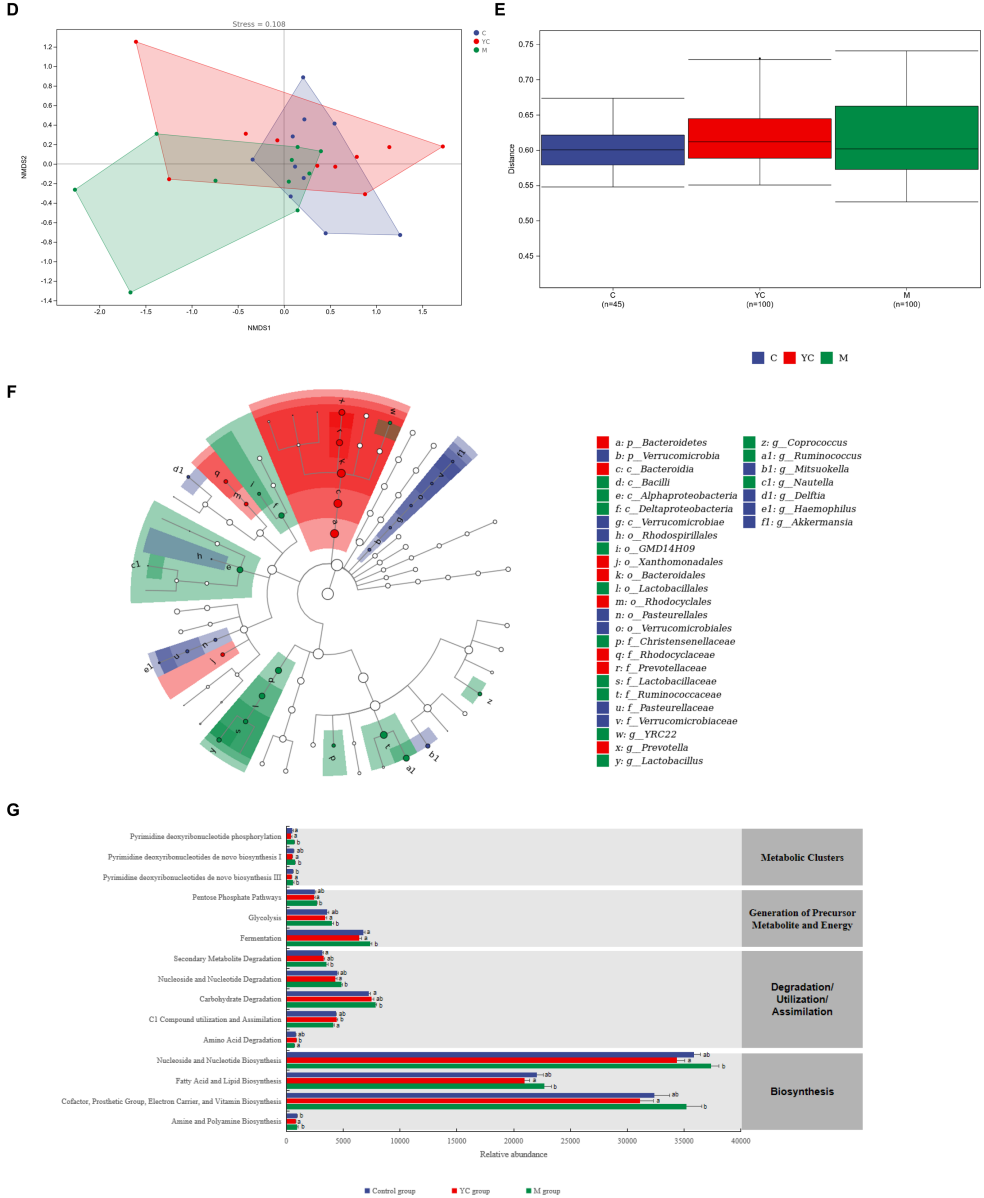
Effects of combined yeast culture (YC) and pure chemicals (M) on ruminal bacterial communities of lambs. **(A)** Alpha diversity of each group; **(B)** Microbiota taxonomic profiling of rumen microbiota at the phylum and **(C)** genus levels; **(D)** non-metric multidimensional scaling analysis (NMDS) and **(E)** analysis of similarity (ANOSIM); Analysis of **(F)** linear discriminant analysis effect size (LEfSe) and **(G)** the relative abundance of bacterial KEGG level 2 in rumen. C, control; YC, yeast culture; M, pure chemicals combination.

A total of 2,073,894 high-quality 16S rDNA gene sequences were obtained from rumen fluid samples. The diet supplementation combining YC and pure chemicals had no significant impact on Chao 1, Faith_pd, Shannon, and Simpson index values (*p* > 0.05, Figure 6A). The top 10 predominant phyla are shown in Figure 6B. Bacteroidetes, Firmicutes, Proteobacteria, and Actinobacteria were present in all animal rumens with relative richness >1%. Cyanobacteria, a less abundant phylum, had a relative average richness of 0.064% and a presence rate of 66% in test animals. Bacteroidetes and Firmicutes were the most abundant in each group. Bacteroidetes was significantly more abundant in the YC group than the M group (*p* < 0.05). In the control and YC groups, Bacteroidetes was more abundant than Firmicutes, but the opposite was true in the M group. The M group had a significantly higher Firmicutes/Bacteroidetes (F/B) ratio than the YC group (*p* < 0.05).

The top 20 predominant genera are shown in Figure 6C. The relative abundance of *Prevotella* was highest in each group, followed by *Shuttleworthia, Butyrivibrio, Ruminococcus, Selenomonas, Coprococcus, Dialister, Succiniclasticum,* and *Succinivibrio.* In the M group, *Prevotella* abundance was lower than control and YC (*p* < 0.05). *Ruminococcus* and *Coprococcus* richness was highest in M, significantly higher than YC (*p* < 0.05). *Megasphaera* and *Mitsuokella* richness decreased with YC and pure chemicals, with *Megasphaera* in YC significantly lower than control (*p* < 0.05). *Mitsuokella* richness in YC and M was significantly lower than control (*p* < 0.05).

The samples of the control group were distributed intensively in the NMDS (Figure 6D), while the samples of the YC group and the M group were relatively far away from those of the control group. The results indicate that dietary supplementation combining YC and pure chemicals had a great effect on rumen microorganisms. In addition, ANOSIM (Figure 6E) showed that the similarities among the treatments were greater than the similarities within the treatments; however, the similarities among the treatments were not significant (R = 0.049, *p* = 0.078).

Figure 6F shows the significantly different bacterial species in each treatment group. The relative abundances of 11 bacterial species showed significant differences in the control group (LDA > 2, *p* < 0.05). The relative abundance of Verrucomicrobia at the phylum level and *Mitsuokella*, *Delftia*, *Haemophilus,* and *Akkermansia* at the genus level were significantly higher than those in the YC and M groups (*p* < 0.05). The relative abundances of eight bacterial species showed significant differences in the YC group (LDA > 2, *p* < 0.05). The relative abundance of *Prevotella* in Bacteroidetes was significantly higher than that in the control and M groups (*p* < 0.05). The relative abundances of 13 bacterial species showed significant differences in the M group (LDA > 2, *p* < 0.05). The relative abundances of *Ruminococcus*, *Lactobacillus,* and *Coprococcus* in Firmicutes and *YRC22* in Bacteroidetes and *Nautella* in Proteobacteria were significantly higher than those in the control and YC groups(*p* < 0.05).

To assess the potential functions, we compared the differences among the three treatments (Figure 6G). At KEGG level 2, 14 differential metabolic pathways belonged to four metabolic pathways of KEGG level 1: biosynthesis, degradation/ utilization/ assimilation, generation of precursor metabolite and energy, and metabolic clusters. The relative abundance of cofactor, prosthetic group, electron carrier, vitamin, fatty acid, nucleoside and nucleotide biosynthesis, glycolysis, and pentose phosphate pathways were higher in M group than YC group (*p* < 0.05). Amino acid degradation, C1 compound utilization and assimilation were higher in YC group than control and M groups (*p* < 0.05). Carbohydrate and secondary metabolite degradation were higher in YC group than control group (*p* < 0.05).

#### 3.2.5 Rumen metabolites

**Figure 7:**
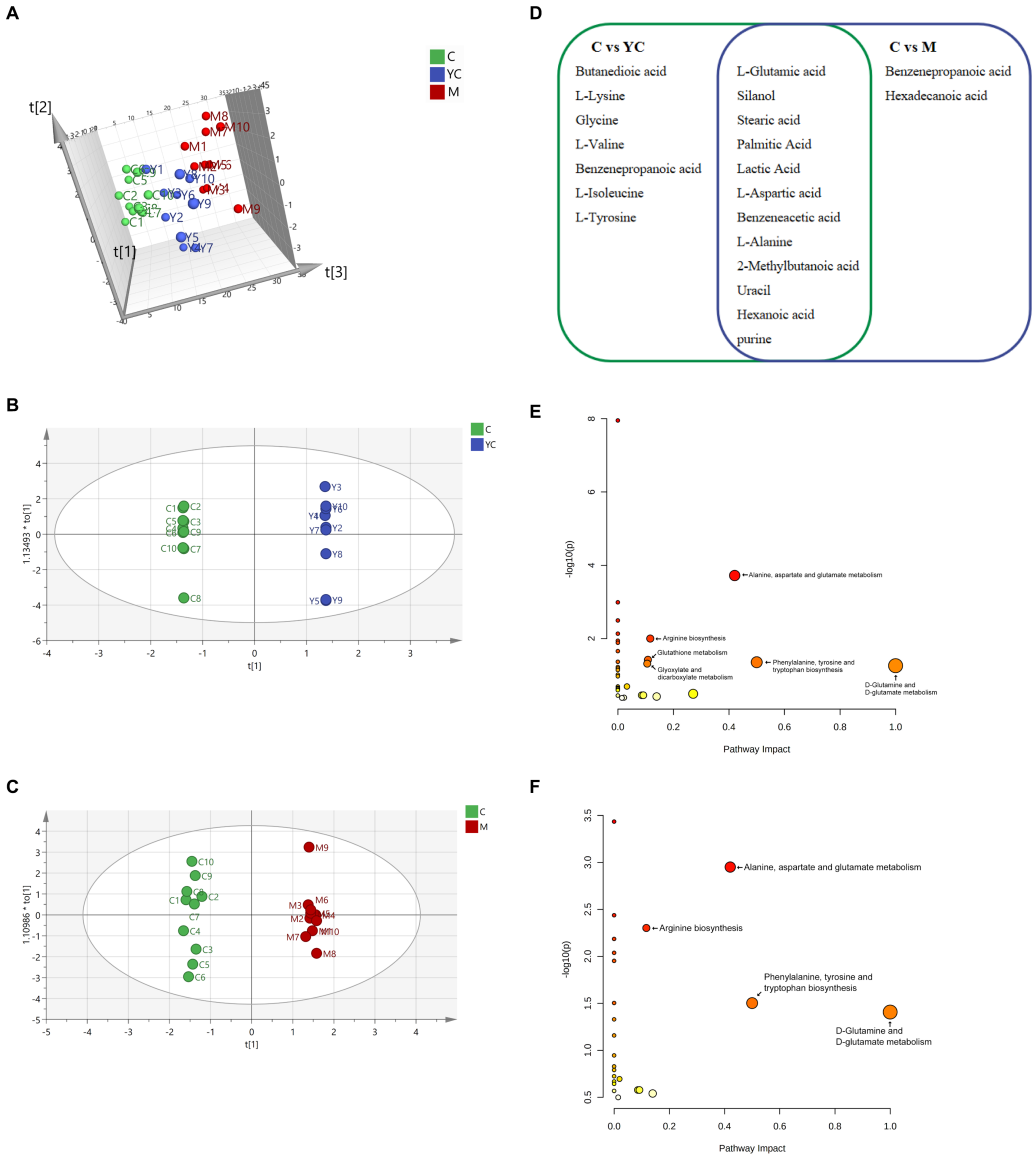
Effect of combined yeast culture (YC) and pure chemicals (M) on rumen metabolites. **(A)** 3-D principal component analysis (PCA) score map of each group and orthogonal partial least squares-discriminate analysis (OPLS-DA) score plots in **(B)** control vs. YC group and **(C)** control vs. M group. **(D)** Different metabolites in control vs. YC group and control vs. M group. Enrichment of metabolic pathways of different substances in **(E)** control vs. YC group and **(F)** control vs. M group. C, control; YC, yeast culture; M, pure chemicals combination.

A total of 119 metabolites of rumen fluid samples in the three treatments were identified using GC/MS according to a matching similarity >800. The 3D-PCA analysis (Figure 7A) revealed clustering of parallel samples within treatment groups, but some mixing across groups. Nevertheless, some sample overlap suggested similar microbial metabolism in rumen fluid of sheep fed YC or pure chemicals. To explore differential metabolites, OPLS-DA was conducted comparing control vs. YC and control vs. M groups. The microbial metabolism in the control and YC groups is significantly separated in Figure 7B. According to the effectiveness evaluation of the model, the analysis showed that R2X = 0.45, R2Y = 1, and Q2 0.464. Generally, Q2 > 0.5 was considered to be a good predictor of the model; however, owing to individual differences in the test animals, Q2 > 0.4 was acceptable. Thus, diet supplementation with YC had a significant impact on microbial metabolism in the rumen fluid of sheep. Similarly, Figure 7C shows the separation of microbial metabolism in the control and M groups; here, the effectiveness evaluation of the model showed that R2X = 0.45, R2Y = 1, and Q2 = 0.464. These results indicate that diet supplementation with pure chemicals combination significantly changed the microbial metabolism in the rumen fluid of sheep.

A total of 21 metabolic candidates (VIP > 1, *p* < 0.05) were obtained between the control and YC groups and between the control and M groups (Figure 7D). Silanol, 1-alanine, uracil, purine, and l-glutamic, stearic, palmitic, lactic, l-aspartic, acetic, benzeneacetic acid, 2-methylbutanoic, and hexanoic acids, were the common metabolic candidates of control vs. YC group and control vs. M group. Compared with the control group, a total of 19 metabolic candidates were identified in the YC group, and 14 metabolic candidates were identified in the M group, which were mainly amino acids and organic acids. The relative concentrations of metabolic candidates in the YC and M groups were significantly higher than those in the control group (*p* < 0.05).

The control and YC groups’ metabolic candidates enriched 33 pathways. Alanine, aspartate, glutamate metabolism, arginine biosynthesis, phenylalanine, tyrosine, tryptophan biosynthesis, d-glutamine, d-glutamate metabolism, glutathione metabolism, and glyoxylate, dicarboxylate metabolism were significant (*p* < 0.05, impact >0) (Figure 7E). The control and M groups’ metabolic candidates enriched 25 pathways. Alanine, aspartate, glutamate metabolism, arginine biosynthesis, phenylalanine, tyrosine, tryptophan biosynthesis, and d-glutamine, d-glutamate metabolism were significant (*p* < 0.05, impact >0) (Figure 7F). The significant metabolic pathways in milk were primarily involved in three KEGG level 2 metabolic pathways: amino acid metabolism, metabolism of other amino acids, and carbohydrate metabolism (Table 5).

**Table 5:** Primary metabolic pathways.

KEGG level 2	Pathway name	Metabolites	Treatment	*p*-value	Impact value
Amino acid metabolism	Alanine, aspartate and glutamate metabolism	L-Aspartate, L-Alanine, L-Glutamate, Succinate	Control vs. YC control vs. M	<0.01	0.42
	Arginine biosynthesis	L-Glutamate, L-Aspartate	Control vs. YC control vs. M	<0.01	0.12
	Phenylalanine, tyrosine and tryptophan biosynthesis	L-Tyrosine	Control vs. YC control vs. M	<0.05	0.50
Metabolism of other amino acids	D-Glutamine and D-glutamate metabolism	L-Glutamate	Control vs. YC Control vs. M	<0.05	1
	Glutathione metabolism	Glycine, L-Glutamate	Control vs. YC	<0.01	0.11
Carbohydrate metabolism	Glyoxylate and dicarboxylate metabolism	Glycine, L-Glutamate	Control vs. YC	<0.05	0.11

YC, yeast culture; M, pure chemicals combination.

**Figure 8:**
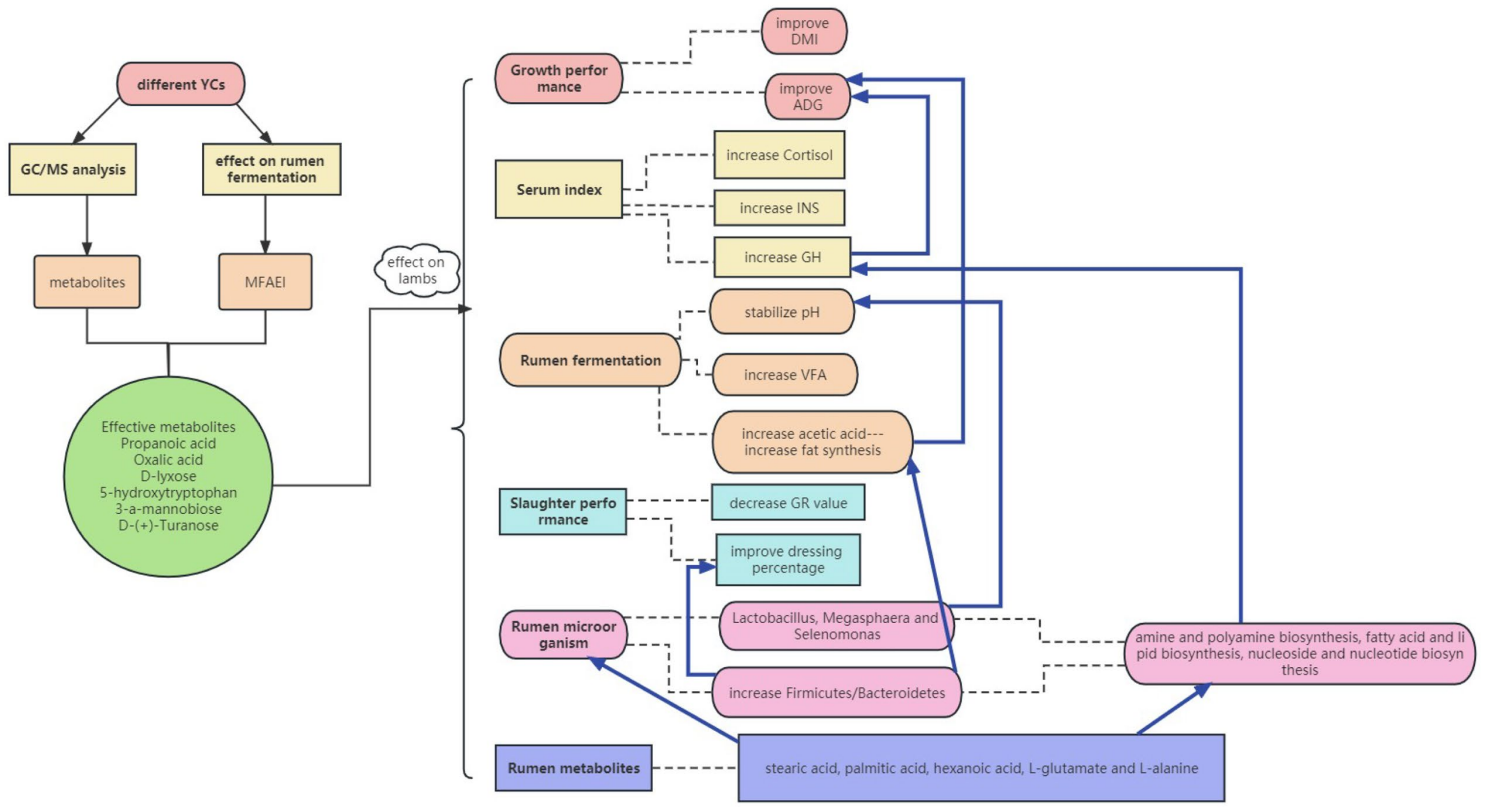
Mechanistic insights into the rumen function promotion by yeast metabolites using *in vitro* and *in vivo* models.

The overall results of the mechanistic insights into rumen function promotion through yeast metabolites are shown in Figure 8. Validation of the production tests in lambs showed that the screened effective substances in YC had positive effects on animal growth performance, slaughter performance, rumen fermentation, and microbial metabolism. The pure chemicals combination promoted the up-regulation of rumen metabolites in the amino acid and carbohydrate metabolism pathways by promoting rumen microbial fermentation. In addition, pure chemicals combination increased serum GH and INS concentrations by directly or indirectly changing the metabolic mechanisms in lambs.

## 4 Discussion

### 4.1 Trial 1: metabolites of different yeast cultures

pH, NH3-N, VFA, MCP and DMD are important parameters to evaluate rumen function *in vitro*. NH3-N is an important index of rumen nitrogen metabolism, microbial protein synthesis, and protein degradation in feed. The proper concentration of NH3-N is a prerequisite to ensure the synthesis of MCP in the rumen. VFA is the main energy source for the maintenance and growth of rumen microbial (Yuan and Wan, 2019). Rumen fermentation parameters *in vitro* were improved by different YC groups. However it may be inaccurate to measure the effect of different YC on rumen fermentation *in vitro* by means of DMD, NH3-N, MCP or VFA alone or several indices because of the complexity of the rumen fermentation mechanism. In this experiment, MFAEI of group B (YC-B) was optimal for rumen fermentation at 6 h, and different YCs had different MFAEI on rumen fermentation *in vitro*, which may be due to the differences in the composition and proportion of efficacy substances in different YCs.

Propanoic acid is one of the main metabolites produced in YC (Kondo et al., 2014), and the production of oxalic acid was first reported by García-Fraile et al. (2013). In the present study, the concentrations of oxalic acid and propionate decreased with the extension of the YC anaerobic fermentation time. This decrease may be attributed to yeast cells entering senescence and apoptosis stages. During this production process, nutrients were no longer supplied to yeast after anaerobic fermentation for 12 h. Nutrient scarcity in the yeast growth environment led to the production of harmful secondary metabolites, affecting the metabolic enzyme system of yeast cells. This resulted in greater decomposition of organic acids than synthesis, with many organic acids constantly transformed and consumed as carbon metabolism substrates. This may also be the cause of the decrease in oxalic and propionic acid concentrations.

Kondo et al. (2014) detected d(−)-lyxose, turanose, and mannose in yeast culture metabolites through GC-accurate mass-ToFMS. There are few reports on the effects of d(−)-lyxose. Turanose, which is naturally found in honey, displays one-half of the sweetness of sucrose, and has been reported to prevent obesity and related metabolic diseases through its anti-inflammatory and adipogenesis-suppressing properties (Park et al., 2016). In this experiment, the concentration of turanose decreased with the extension of the YC anaerobic fermentation time, which may be because turanose is used by yeast during anaerobic fermentation. Mannan is found in the outermost layer of the yeast cell wall and is composed of α-1,6 bonds as the backbone chain, with the side chains consisting of mannose units connected to the backbone with α-1,2 bonds (Halász and Lasztity, 2017). Interestingly, mannan in YC can improve the intestinal environment and enhance the immune and antioxidant functions of animals (Borovikova et al., 2016; Korolenko et al., 2020).

Zhang et al. (2016) reported that 5-hydroxytryptophan can be synthesized from *S. cerevisiae*. Furthermore, 5-hydroxytryptophan is a serotonin precursor that is used to treat anxiety, depression, insomnia, and obesity (Nash and Jamshidi, 2022). Valente et al. (2021) reported that administering moderate doses of 5-hydroxytryptophan to the abomasum increases serotonin synthesis and feed intake. However, there are few reports on the effects of 5-hydroxytryptophan on rumen fermentation, which need to be further explored.

The microbial fermentation environment is extremely complex, and metabolites produced by yeast cells may be used by yeast cells as nutrients and absorbed into cells. The utilization, transformation, and generation of metabolites in yeast cells are dynamic processes. Therefore, the metabolites of the YCs monitored in this experiment only represent the results of the analysis under specific experimental conditions.

### 4.2 Trial 2: growth and slaughter performance of lambs

Study on the effects of YC supplementation on ADG were different. Haddad and Goussous (2005) showed that YC can significantly improve ADG and feed conversion in lambs. Moreover, Soren et al. (2013) reported that the ADG was not significantly influenced by YC supplementation in lambs. Abu El-Kassim et al. (2021) also reported that YC improved the ADG of lambs, although the increase did not reach statistical significance, consistent with the findings of the present study. In this study, supplementation with YC and pure chemical combinations improved the ADG of lambs, which may be because of the presence of mannan and 5-hydroxytryptophan in both YC and pure chemical combinations. Polysaccharides such as mannan and other active substances in YC can promote rumen fermentation and intestinal microbial metabolism in ruminants, thereby improving animal performance. Moreover, 5-hydroxytryptophan (5-HTP) is an intermediate of serotonin biosynthesis (Niu, 2011). The neurotransmitter serotonin is thought to produce feelings of calmness, relaxation, and contentment, which can promote sleep in animals and reduce their maintenance needs, thereby improving their feed utilization efficiency and growth performance. Liu et al. (2019a) previously reported that dietary supplementation with YC increased the dressing percentage of lambs, aligning with the findings of this study. In our experiment, YC supplementation improved the dressing percentage of lambs, with the M group displaying the highest dressing percentage. The growth-promoting effect of pure chemical combinations may be because of the reduced abdominal fat percentage, thus saving energy for muscle growth. Simultaneously, back fat thickness in both the YC and M groups showed a decreasing trend, suggesting that YC and the pure chemical combination may primarily regulate muscle fat deposition. However, the specific regulatory mechanism warrants further investigation. Notably, the dressing percentage in the M group was higher than that of the YC group. This difference could be attributed to the pure chemical combination containing only substances from YC that positively affected rumen fermentation, while potentially excluding negative substances. The antagonistic effects of these negative substances in YC on rumen regulation may indirectly influence growth performance, resulting in a less favorable effect of YC on the dressing percentage of lambs compared to the pure chemical combination.

### 4.3 Serum index of lambs

Cortisol, an acute stress hormone and nonspecific stress indicator, regulates metabolism, including lipolysis, gluconeogenesis, and glycogenosynthesis (Yamaji et al., 2009; Arfuso et al., 2023). In this study, cortisol levels rose in the YC and M groups, possibly due to a moderate cortisol increase that can enhance growth in non-stressed animals. GH is secreted by the hypothalamus and plays a crucial role in animal growth, promoting the growth of body tissues, elevating circulating glucose levels, and facilitating protein and fat breakdown (Qi et al., 2017). Insulin reduces blood glucose levels and promotes lipogenesis in tissues (Wang et al., 2021). In the present study, there was no significant difference in serum glucose levels among the treatment groups, indicating that GH and INS levels in lambs were balanced. Moreover, the levels of GH and INS in the YC and M groups were higher than those in the control group. This is consistent with the findings of Murakami et al. (1986), who observed a significant increase in plasma GH levels in rats following intravenous injection of 5-HTP or intracerebroventricular injection of serotonin. This may be because 5-HTP in YC and the pure chemical combination promote the synthesis of 5-HT and stimulate the secretion of GH. When GH levels rise, blood glucose levels also increase, leading to an increase in INS, maintaining stable blood glucose levels. LEP, a peptide hormone secreted by adipocytes, is crucial in regulating diet, fat, and energy metabolism (Morton et al., 2005 Khan et al., 2012). On day 30, serum LEP levels significantly rose in lambs from the YC and M groups. However, on day 60, there was no significant difference in LEP levels among groups, indicating no significant relationship between body weight and serum LEP levels. Serum TP, which mainly includes ALB and globulin, is an indicator of the nutritional status of animal (Doornenbal et al., 1988). Abu El-Kassim et al. (2021) reported that dietary yeast supplementation increased blood plasma ALB concentrations in Ossimi lambs, which was consistent with the results of the current study. This indicates that the serum concentrations of ALB were affected by YC and pure chemical combination supplementation, possibly resulting from the improvement in nutrient digestibility in the feed.

### 4.4 Rumen fermentation parameters of lambs

Ruminal pH and VFAs are important indicators of ruminal function and changes in the ruminal environment. Wang et al. (2023) found positive effects of dietary YC on ruminal pH in sheep; however, the present study observed no significant effects. Consistent with our results, Zhu et al. (2017) reported that feeding YC did not significantly impact rumen pH. VFAs are the final products of carbohydrate fermentation in the rumen, providing nearly 50% of the energy required for microbial synthesis in the rumen (Puniya et al., 2015; Liu et al., 2021). YC supplementation and its influence on ruminal VFAs have led to mixed results (Liu et al., 2019b; Carpinelli et al., 2021). Halfen et al. (2021) evaluated the effect of YC supplementation on ruminal fermentation in mid-lactation dairy cows, and their results suggested that YC increased the total concentrations and decreased the ratio of acetic acid to propionic acid. In contrast, Moya et al. (2009) and Xiao et al. (2016) observed that YC supplementation had no significant effect on ruminal VFA. YC supplementation tended to increase total VFA (especially acetic acid and propionic acid) production, but had no significant effect in the current study. However, acetic acid and propionic acid in the M group significantly increased, which may be due to the combination of pure chemicals containing only the substances in YC that positively regulate rumen fermentation. Thus, the positive effect of the pure chemical combination on rumen VFA concentrations was more significant than that of the YC. Acetic acid is the substrate for fat synthesis (Urrutia and Harvatine, 2017), and its concentration in the M group was the highest, indicating that the lambs tended to synthesize more fat. Furthermore, the pure chemical combination contained propionic acid in it, which can provide energy for rumen fermentation. Although the total VFA concentrationsin the YC and M groups tended to increase, the addition of YC and pure chemicals did not result in a decrease in ruminal pH, which may be due to the absorption of VFAs.

### 4.5 Rumen microorganisms and metabolites

Rumen microorganisms form different microbial communities during the degradation of various dietary components. The NMDS and ANOSIM analyses showed that the structure and composition of ruminal bacteria were altered by the combination of YC and pure chemicals in lambs, although the influence was not significant in the present study. Liu et al. (2019b) and Han et al. (2021) found that alpha diversity increased, and the richness of the rumen bacterial community was increased by YC supplementation in sheep. However, supplementation with YC and pure chemicals did not influence the alpha diversity index of the rumen bacterial communities in the current study. Similar results were reported by Wang et al. (2023), who found that supplementation with YC did not influence bacterial diversity in the rumen of sheep, which was possibly because of differences in animal models, diets, and the level and type of YC supplementation.

The most abundant phylum was *Bacteroidetes,* followed by *Firmicutes* and *Proteobacteria*, which was consistent with previous findings on the rumen microbiota of lambs (Shen et al., 2020; Zhou et al., 2022). *Firmicutes* play an important role in the degradation of decomposed cellulose, starch, and oligosaccharides and are tightly associated with energy utilization and growth performance (Han et al., 2020; Zhou et al., 2022). *Bacteroidetes* can degrade both carbohydrates and proteins (Lopes et al., 2015). Moreover, *Firmicutes* and *Bacteroidetes* have a symbiotic relationship, and obesity is associated with the abundance of these two bacteria. Ley et al. (2006) reported that the relative abundance of *Bacteroidetes* in people with obesity was lower than that in lean people, with the ratio of *Firmicutes* and *Bacteroidetes* being particularly important. Moreover, the higher the relative richness ratio between *Firmicutes* and *Bacteroidetes* (*Firmicutes/Bacteroidetes*), the stronger the lipid deposition capacity of animals (Bäckhed et al., 2004). Turnbaugh et al. (2006) found that the gut microbiome associated with obesity has an enhanced ability to derive energy from the diet. Similarly, the *Firmicutes/Bacteroidetes* value of the M group was higher than those of the control and YC groups, which might explain why the body weight and dressing percentage of lambs in the M group were higher than those in the control and YC groups.

*Prevotella*, the dominant bacterium in the YC group, plays a role in carbohydrate utilization, nitrogen metabolism, and fiber degradation (Li et al., 2020; Xiong et al., 2023). It belongs to the phylum *Bacteroidetes* and is the core bacterium with the highest richness in the rumen (Henderson et al., 2015). Consistent with our findings, Wang et al. (2023) found that *Prevotella* decreases with YC supplementation. Liu et al. (2019b) observed a decline in *Prevotella* in the rumens of lambs 6 h after consuming a high non-structural carbohydrate to fat ratio diet with YC, followed by an increase when fed a low ratio diet. These results indicate that rumen microorganisms vary with different diets and that the effects of YC on rumen microorganisms are related to diet. The relative abundance of *Ruminococcus* and *Lactobacillus* was highest in the M group. *Ruminococcus* plays an important role in cellulose degradation in the rumen (Falalyeyeva et al., 2022). The carbohydrate metabolism pathway consistently exhibited higher levels in the M group than in the control group. *Lactobacillus,* a gram-positive bacterium that primarily produces lactic acid during fermentation (Ibrahim, 2016), can lead to SARA when it becomes the dominant bacteria in the rumen. Moreover, *Megasphaera* and *Selenomonas,* lactate-consuming bacteria in the rumen (Wang et al., 2014), were detected in all treatments and belonged to the top 20 predominant genera. The relative abundance of *Lactobacillus* and lactate-consuming bacteria was within the normal range, and rumen microorganisms were in a balanced state to prevent rumen acidosis.

This study highlights the key metabolic pathways in ruminal microorganisms, including biosynthesis, degradation, utilization, assimilation, and precursor metabolite/energy generation. Biosynthesis is the most common, assisting in dietary nutrient digestion and ruminant energy/nutrient provision. In the M group, metabolic pathways related to amine/polyamine, fatty acid/lipid, and nucleoside/nucleotide biosynthesis were more abundant than in the control and YC groups. This suggests that M group microorganisms were more efficient in generating polyamines and fatty acids from intermediates, boosting ruminant growth energy. This study confirmed that dietary supplementation with YC and pure chemical combinations resulted in different metabolites in ruminal fluid, both of which promoted the synthesis of fatty acids such as stearic acid, palmitic acid, and hexanoic acid, and amino acids such as l-glutamic acid, l-alanine, and benzeneacetic acid in the rumen. Moreover, stearic acid and palmitic acid are saturated fatty acids. Kemp and Lander (1984) reported that unsaturated fatty acids can be detrimental to rumen microorganisms, potentially inhibiting their growth. In response, microorganisms may hydrogenate unsaturated fatty acids into saturated fatty acids as a metabolic detoxification process, a form of self-protection. The current findings suggest that the supplementation of YC and pure chemical combinations in the diet may promote the hydrogenation of rumen microorganisms. Amin and Onodera (1997) reported that phenylalanine, benzoic acid, and phenylpropionic acid are produced from benzeneacetic acid in the presence of ruminal microbes. The addition of YC and pure chemical combinations increased the concentration of benzeneacetic acid in rumen microbial metabolites in the present study (Urrutia and Harvatine, 2017). In addition, the increased L-glutamate and L-alanine in the YC and M groups can provide substrates for the synthesis of ruminal microorganisms. The metabolic candidates identified in the YC and M groups mainly enhance amino acid and carbohydrate metabolism pathways. This increase in carbohydrates could provide additional energy for the synthesis of MCP.

## 5 Conclusion

Propanoic acid, oxalic acid, D-lyxose, 5-hydroxytryptophan, 3-a-mannobiose and D-(+)-turanose were preliminarily identified as the main YC effective substances *in vitro*. These substances positively affects the slaughter performance, rumen fermentation, and microbial metabolism *in vivo*. Additionally, the effectiveness of these substances (pure chemical combinations) was superior to that of YC in improving the slaughter performance of lambs. This study not only gives technical parameters for optimizing the quality and efficacy of YC, but also provides some theoretical base for further research on safe and effective feed additives.

## Data availability statement

The NCBI Sequence Read Archive (SRA) database contains the raw sequence reads for all samples described in the study (Accession No. PRJNA1024379).

## Ethics statement

The animal studies were approved by the Institutional Animal Care Committee of Jilin Agricultural University (JLAU – A CUC2021-013) approved the comprehensive process used in the management of the experimental animals. The studies were conducted in accordance with the local legislation and institutional requirements. Written informed consent was obtained from the owners for the participation of their animals in this study.

## Author contributions

XC: Writing – original draft. JX: Data curation, Formal analysis, Writing – original draft. WaZ: Data curation, Writing – original draft. YL: Formal analysis, Writing – original draft. WeZ: Data curation, Writing – original draft. WGZ: Formal analysis, Writing – original draft. LX: Data curation, Writing – original draft. ZH: Formal analysis, Writing – original draft. LW: Formal analysis, Writing – original draft. NA: Formal analysis, Writing – review & editing. XZ: Methodology, Writing – review & editing. TW: Writing – original draft, Writing – review & editing. GQ: Methodology, Writing – review & editing. ZS: Writing – review & editing, Writing – original draft. YZ: Writing – review & editing.

## Footnotes

^1^ https://docs.qiime2.org/

^2^ http://www.metaboanalyst.ca
